# Contrast‐enhanced ultrasound and photoacoustic imaging to assess early and late renal ischemia‐reperfusion injuries in male mice

**DOI:** 10.14814/phy2.70973

**Published:** 2026-06-18

**Authors:** Maxime Schleef, Corentin Tournebize, Christelle Leon, Bruno Pillot, Stéphanie Chanon, Gabriel Bidaux, Laurent Juillard, Fitsum Guebre‐Egziabher, Delphine Baetz, Sandrine Lemoine

**Affiliations:** ^1^ Nephrology‐Dialysis‐Renal Functional Explorations Edouard Herriot Hospital, Hospices Civils of Lyon Lyon France; ^2^ Université Lyon 1, CarMeN Laboratory, INSERM, INRAE Université Lyon 1 Claude Bernard Bron France

**Keywords:** contrast‐enhanced ultrasound, fibrosis, ischemia‐reperfusion, perfusion, photoacoustic imaging

## Abstract

Tissue perfusion and oxygenation, key factors of renal ischemia‐reperfusion injury (RIR), can be assessed using contrast‐enhanced ultrasound (CEUS) and photoacoustic (PA) imaging. We hypothesized that early changes in renal perfusion and oxygenation caused by IRI, or protection by mild therapeutic hypothermia (mTH), could be detected and correlate with fibrosis. C57BL6 mice underwent 15‐min unilateral renal ischemia, with or without mTH, followed by 1‐month reperfusion. They were assigned to sham (*n* = 5), normothermic renal ischemia (IR‐37°C, *n* = 7), mTH renal ischemia (IR‐34°C, *n* = 7). The IR‐37°C and IR‐34°C groups had body temperature maintained at 37°C or 34°C during surgery. CEUS and PA were conducted at baseline, 20 min, and 1 month after reperfusion. Histological analysis was performed at 1 month. IRI induced renal atrophy and fibrosis at 1 month, prevented by mTH. CEUS revealed altered perfusion in IR‐37°C at 20 min, sustained at 1 month, compared to Sham, while perfusion was preserved with mTH in IR‐34°C. Oxygenation assessed by PA was not modified. However, oxygenation measured 20 min after IRI correlated with fibrosis at 1 month. CEUS and PA are promising tools for non‐invasive assessment of renal IRI. IRI induced early and sustained perfusion alterations, while early assessment of oxygenation correlated with fibrosis. mTH prevented these alterations.

## 1 INTRODUCTION

Renal ischemia‐reperfusion injury (IRI) is a major cause of acute kidney injury (AKI) (Lameire et al., 2008). Tissue repair following AKI can be incomplete and maladaptive, leading to the development of irreversible fibrosis and chronic kidney disease (CKD) (Basile et al., 2016; Coca et al., 2009). Early biomarkers of AKI are lacking. Serum creatinine level is a late and indirect biomarker, increasing only when a significant filtration capacity has been lost and kidney damage is advanced (Zhang & Parikh, 2019). Tissue perfusion and oxygen content are two major determinants of IRI but are not usually recorded because they are challenging to measure either in experimental set‐up or in clinical practice in the kidney (Fine et al., 1998). However, because the early detection of IRI is essential for preventing CKD and the development of irreversible interstitial fibrosis, these parameters are highly relevant to quantify.

Ultrasound imaging is a widely accessible and valuable diagnostic tool, offering real‐time imaging with good spatial resolution, low cost, and the advantages of bedside application, absence of ionizing radiation, and minimal risk of adverse effects. Emerging ultrasound techniques, such as contrast‐enhanced ultrasound (CEUS) and photoacoustic (PA) imaging, enable the evaluation of renal perfusion and oxygenation, offering new insights into renal function (Kruger, 1994; Wei et al., 2001). CEUS quantifies renal blood flow by intravenous administration of microbubbles, a strictly intravascular contrast agent, and has been identified as an early predictor of acute and chronic renal lesions in AKI, especially in rodent IRI models (Correas et al., 2001; Sun et al., 2020). PA imaging is a promising functional imaging modality by combining optical contrast with the spatial resolution of ultrasound. This hybrid technique capitalizes on the photoacoustic effect, where pulsed laser light is absorbed by tissue chromophores—predominantly oxygenated and deoxygenated hemoglobin—resulting in the emission of acoustic waves that are subsequently detected and reconstructed into high‐resolution images (Rich & Seshadri, 2015). Previous publications have underscored the potential of PA to noninvasively assess renal oxygenation. Notably, PA can detect subtle alterations in tissue oxygen saturation, potentially serving as an early biomarker for AKI (Meyer et al., 2021).

In this experimental study, we hypothesized that early and late alterations of renal perfusion and oxygen content after renal IRI could be measured with CEUS and PA imaging. Based on previous results demonstrating the protective effects of mild therapeutic hypothermia (mTH) during renal ischemia (Schleef et al., 2022), we repeated this intervention in the same model. We also hypothesized that functional imaging parameters assessed early with CEUS and PA would correlate with the extent of subsequent renal fibrosis in the settings of IRI with or without nephroprotective intervention.

## 2 METHODS

### 2.1 Animals and surgical procedure

Eight‐ to ten‐week‐old male C57BL6 mice (Charles River, France) were studied. We focused only on male mice because of the potentially protective effect of estrogens, to ensure better comparability between the animals and therefore reduce the total number of animals needed, overcoming the difference between sexes. Animals were housed in stable groups of four in individually ventilated cages (Nextgen—Allentown, USA—conventional animal facility) with standard nesting materials (cotton, tunnel) and ad libitum access to filtered water and standard diet (2018 global rodent diet, Envigo, France). Room temperature (housing and experiment) was maintained at 22°C ± 2°C and light cycle was at 12:12. Mice were anesthetized by intraperitoneal injection of xylazine (5 mg/kg, Rompun; Bayer, Puteaux, France), ketamine (100 mg/kg, Imalgene 1000; Acyon, France), and buprenorphine (0.075 mg/kg, Vetergesic; Sogeval, Laval, France). The animals were intubated and ventilated (Minivent, Harvard Apparatus, March, Germany) to perform the procedures described thereafter. Core body temperature was maintained at 37°C or 34°C during the entire surgery using a rectal thermometer and a homeothermic pallet unit (PhysioSuite® Kent Scientific, Torrington, CT, USA). After 1 month, following the completion of the last renal imaging, mice were killed by cervical dislocation under general anesthesia and kidneys were harvested and stored in formaldehyde. All animal procedures were approved by the Ethics Committee (Claude Bernard Lyon 1 University, CEEA‐55, no DR2019‐09) and conducted in accordance with French and European Law.

### 2.2 Experimental groups

Mice were randomly assigned to three groups: sham (*n* = 5), normothermic renal ischemia (IR‐37°C, *n* = 7), and mTH renal ischemia (IR‐34°C, *n* = 7).

**FIGURE 1:**
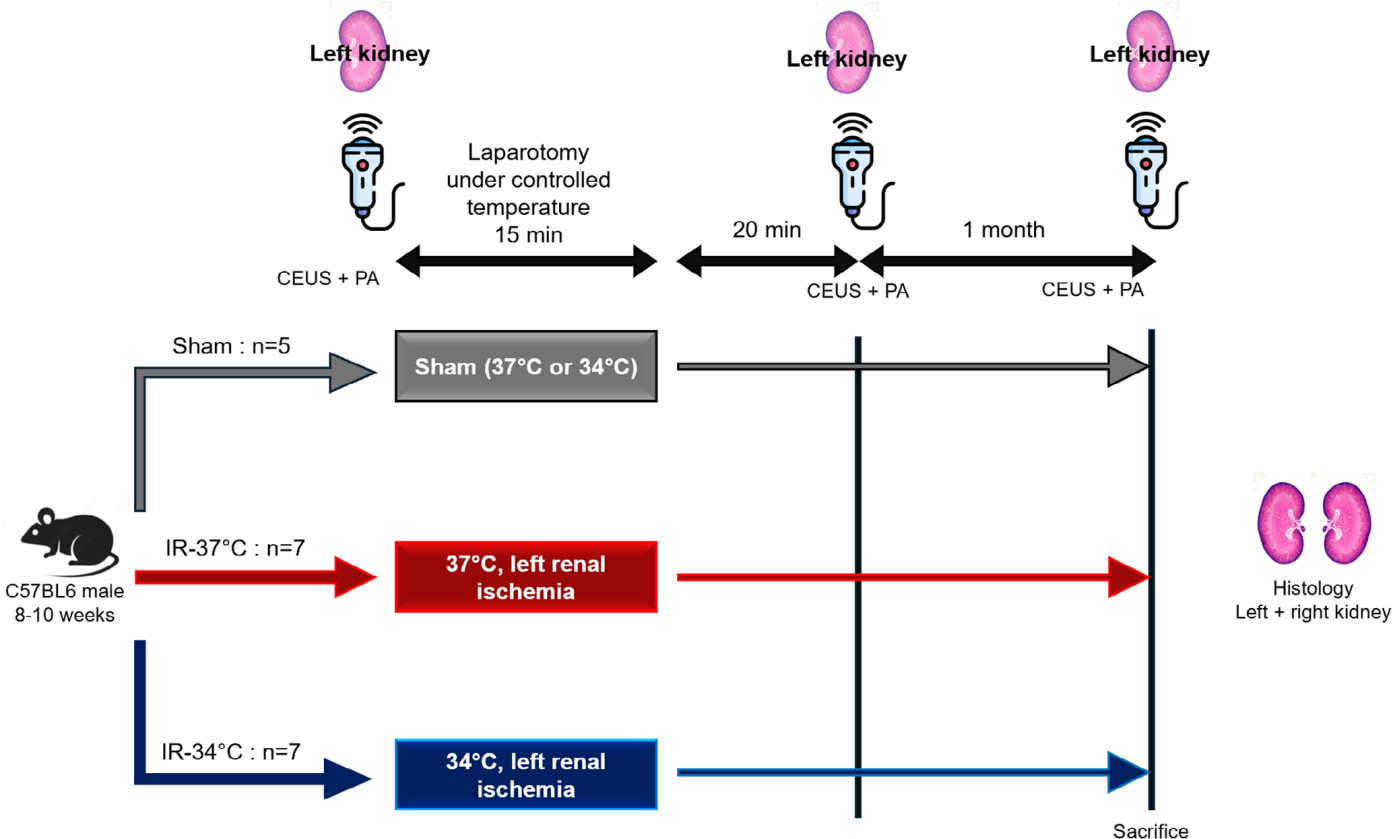
Experimental design.

The IR‐37°C and IR‐34°C groups underwent 15 min of unilateral renal ischemia by selective clamping of the left vascular pedicle using a microvascular clamp (Roboz Surgical Instruments, Washington, DC, USA). Ischemia and reperfusion were confirmed visually with the coloration of the kidneys (Figure 1).

The sham group consisted of a mix of mice undergoing the laparotomy alone, with body temperature maintained at 37°C (*n* = 3) or 34°C (*n* = 2). This was scientifically supported by previous data in the same model where we showed in sham that body temperature, that is, mTH compared to normothermia, alone has no effect on any of the following renal parameters: AKI, acute tubular necrosis, kidney atrophy and fibrosis, and renal vascular resistance (Schleef et al., 2022). The pooling was therefore deemed appropriate, mandated by the ethics committee under the “Reduction” principle of the 3Rs, and necessitated by limited access to CEUS and PA imaging equipment (required for our primary endpoints) in our facility.

### 2.3 Renal histology

The left and right kidneys were fixed in formaldehyde 1 month after reperfusion and embedded in paraffin. To assess renal fibrosis, 4‐μm transversal sections were stained with Masson's trichrome or with Sirius Red. One section was examined for each kidney, and each section was imaged as a whole, enabling the visualization of the whole kidney, rather than focusing only on a few selected fields. Images were analyzed using Fidji software (ImageJ, NIH), with the automatic quantification of fibrosis based on the ratio between green pixels or red pixels (respectively representing collagen, i.e., fibrosis, on Masson's trichrome or Sirius red coloration) and all pixels representing the whole kidney section, expressed as a percentage (Figure S1).

Every visualized and identifiable glomerulus (whether normal/healthy or not) on the whole section was manually delineated by a nephrologist (blinded from group assignment) with QuPath software (v0.5.1 open‐source software). Glomerular surfaces obtained with the software were then averaged for each kidney to obtain mean glomerular surface.

### 2.4 Renal imaging

Renal imaging was conducted under sevoflurane gas anesthesia 1 week before the surgical procedure (basal situation), 20 min after reperfusion, and at 1 month. Renal ultrasound imaging was performed on the left kidney in the supine position using a high‐resolution ultrasonic imaging system (VEVO 3100 Fujifilm Visualsonics, Toronto, Canada) by an experienced operator blinded from the animals' group assignment. An MX550D probe (Fujifilm Visualsonics, Toronto, Canada) was used and fixed in place with an iron support to ensure a constant imaging plane throughout the acquisition. First, regular B‐mode images were used to visualize the entire kidney and the renal artery. A 3D reconstruction was performed through multiplane acquisition with an automatized mobile support to assess the volume of the kidney. Renal CEUS and PA imaging were then performed in a second step, respectively with MX201 and MX550D probes (Fujifilm Visualsonics, Toronto, Canada). Images were recorded and stored for further analysis by two other operators (both nephrologists) who performed the same set of analyses blinded from the animal's assignment and independently from each other. Regions of interest (ROI) were manually placed on each image of the whole kidney (to allow 3D reconstruction), as well as on the cortex, inner medulla, and outer medulla, which could get recognized on B‐mode images.

### 2.5 Renal CEUS

A continuous infusion of microbubbles (VS‐11913, Fujifilm Visualsonics, Toronto, Canada), diluted in serum saline as per manufacturer's instruction, was performed through a venous catheter in the tail vein at a rate of 50 μL/min. After 1–2 min of infusion (around 50–100 μL), once the contrast enhancement had reached a steady state, a high mechanical index burst was applied for 1 s to destroy microbubbles. Then, a low‐mechanical‐index imaging mode was used (on a consistent parasagittal plane with visualization of renal vessels) until 30 s after the contrast agent detection reached a steady plateau again. A second burst was delivered, followed by the same imaging. Overall, a total volume of 250–300 μL was injected for these two acquisitions. Images were recorded and analyzed using the “destruction‐replenishment” fitting model of the Vevo LAB software (Fujifilm Visualsonics, Toronto, Canada). Renal perfusion was estimated using the total relative Blood Flow (rBF) parameter and expressed in arbitrary units. The rBF corresponds to the product of two parameters measured with CEUS: the relative Blood Volume (rBV) in arbitrary units and the mean Transit Time (mTT) in seconds. rBV refers to the plateau value in replenishment kinetics, and mTT measures the average time that blood takes to transit through a portion of the tissue (Figure S2).

### 2.6 Renal PA imaging

Renal oxygen content was measured using PA imaging on multiplane acquisition with an automated support, allowing 3D reconstruction of the kidney and measurements. The 3D oxy‐hemo 750/850 nm mode from the VEVO Lazr‐X module (Fujifilm Visualsonics, Toronto, Canada), of the VEVO 3100 imaging system was used. Images were recorded and analyzed using Vevo LAB software. Renal oxygen content was estimated using the sO2 average‐3D parameter on the whole 3D‐reconstructed kidney, corresponding to the percentage of oxyhemoglobin over the total hemoglobin content (oxyhemoglobin and deoxyhemoglobin) within the ROI (Figure S3).

### 2.7 Statistical analysis

Data are expressed as median with interquartile range (IQR). All groups were compared using the Kruskal–Wallis test. Comparisons between two groups were performed using the Mann–Whitney test. Paired data were compared using Wilcoxon matched‐pairs signed rank tests when appropriate. Correlations between different parameters were tested using Spearman's test. *p* Value less than 0.05 was considered significant. The numbers of animals used were decided by ethical considerations and practical limitations (i.e., availability of the imaging technique) at the time of conductance of the study. Nevertheless, statistical power was calculated after completion of the study, given the negative result on PA imaging parameters. Based on the observed distribution of SO_2_ measured by PA imaging, it was estimated that *n* = 7 in each IR‐group had a 60% power to detect a reduction of 20% (as roughly observed), while it could have detected a 25% reduction during follow‐up with a power of 80%, or a 37.5% reduction with a power of 99%. Statistical analyses were performed using GraphPad Prism v10.2.2 (Graphpad Software, San Diego, CA, USA).

## 3 RESULTS

### 3.1 Renal histology

#### 3.1.1 Fibrosis quantification

**FIGURE 2:**
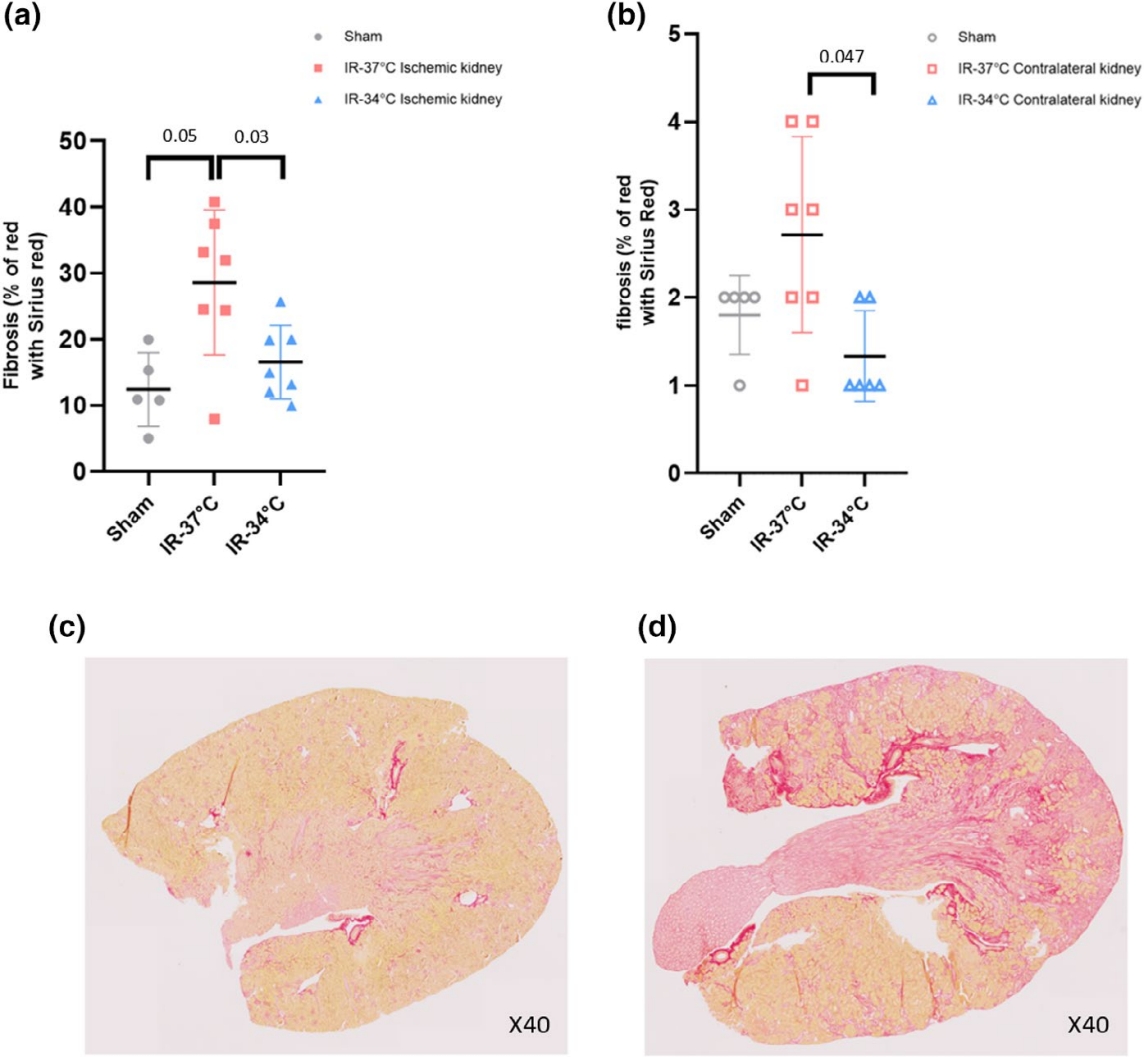
Effect of ischemia‐reperfusion and therapeutic mild hypothermia on kidney fibrosis 1 month after a 15‐min unilateral renal ischemia. (a) Sirius red staining on ischemic kidney, (b) Sirius red staining on the contralateral kidney left untouched during ischemia. Representative examples of a kidney from IR‐34°C with 15% fibrosis (c) and one from IR‐37°C with 41% fibrosis (d) (ratio between red pixels and all pixels) on Sirius red coloration. Data are presented as median with interquartile ranges and compared with Mann–Whitney test.

One month after reperfusion, we detected in the kidney that had undergone ischemia a greater extent of renal fibrosis in the IR‐37°C group, while it was blunted by mTH in the IR‐34°C (Masson's trichrome: 35% IQR [30–47]% vs. 25% IQR [22–27]%; *p* = 0.02; and Sirius red staining: 32% IQR [24–37]% vs. 15% IQR [12–20]%; *p* = 0.03; Figure 2a). Interestingly, some extent of renal fibrosis was also detected in the contralateral kidney (left untouched during ischemia) in the IR‐37°C, which was also attenuated by mTH in the IR‐34°C (Masson's trichrome: 8% IQR [5–14]% vs. 4% IQR [2–9]%; *p* = 0.11; and Sirius red staining: 3% IQR [2–4]% vs. 1% IQR [1–2]%; *p* = 0.047; Figure 2b–d). Renal fibrosis extent in the IR‐34°C was similar to sham, with no significant difference in both the ischemic and contralateral kidney, highlighting the protective effect of mTH in IRI.

#### 3.1.2 Glomerular size

**FIGURE 3:**
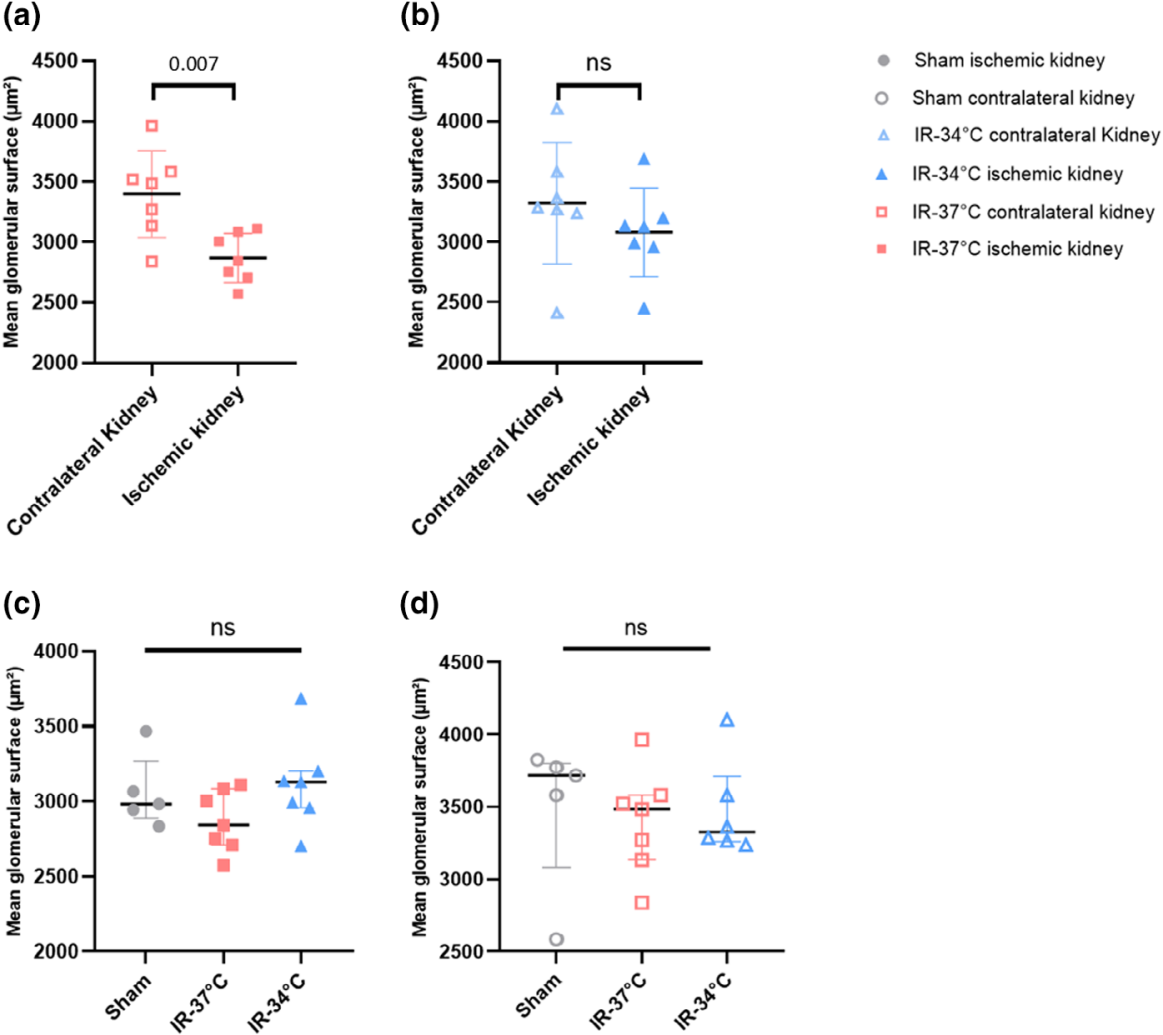
Comparison of kidney's glomerular surface 1 month after reperfusion. (a) Ischemic versus contralateral kidney in IR‐37°C group; (b) Ischemic vs. contralateral kidney in IR‐34°C group; (c) Glomerular surface in ischemic kidneys; (d) Glomerular surface in contralateral kidneys. Data are presented as median with interquartile ranges and compared with Mann–Whitney test.

At 1 month, glomeruli size was significantly smaller in ischemic kidney than in contralateral untouched kidney only in the IR‐37°C group (2834 μm^2^ IQR [2709–3085] μm^2^ vs. 3485 μm^2^ IQR [3134–3582] μm^2^; *p* = 0.007; Figure 3a). On the contrary, no difference was observed between left and right kidney's glomerular size in IR‐34°C group (3287 μm^2^ IQR [3237–3581] μm^2^ vs. 3128 μm^2^ IQR [2958–3202] μm^2^; *p* = 0.13; Figure 3b).

We observed a reduction (although not reaching statistical significance) in glomerular sizes in kidneys that underwent normothermic ischemia (IR‐37°C) compared to kidneys that had undergone mTH ischemia (IR‐34°C) (2843 μm^2^ IQR [2709–3085] μm^2^ vs. 3128μm^2^ IQR [2958–3202] μm^2^; *p* = 0.13; Figure 3c). No difference in glomerular sizes was noted when comparing the untouched contralateral kidneys between them (sham, IR‐37°C, IR‐34°C) (Figure 3d). No significant correlation was found between glomerular size and fibrosis in both ischemic and contralateral kidneys.

### 3.2 Renal ultrasound imaging

#### 3.2.1 Kidney volume quantification

**FIGURE 4:**
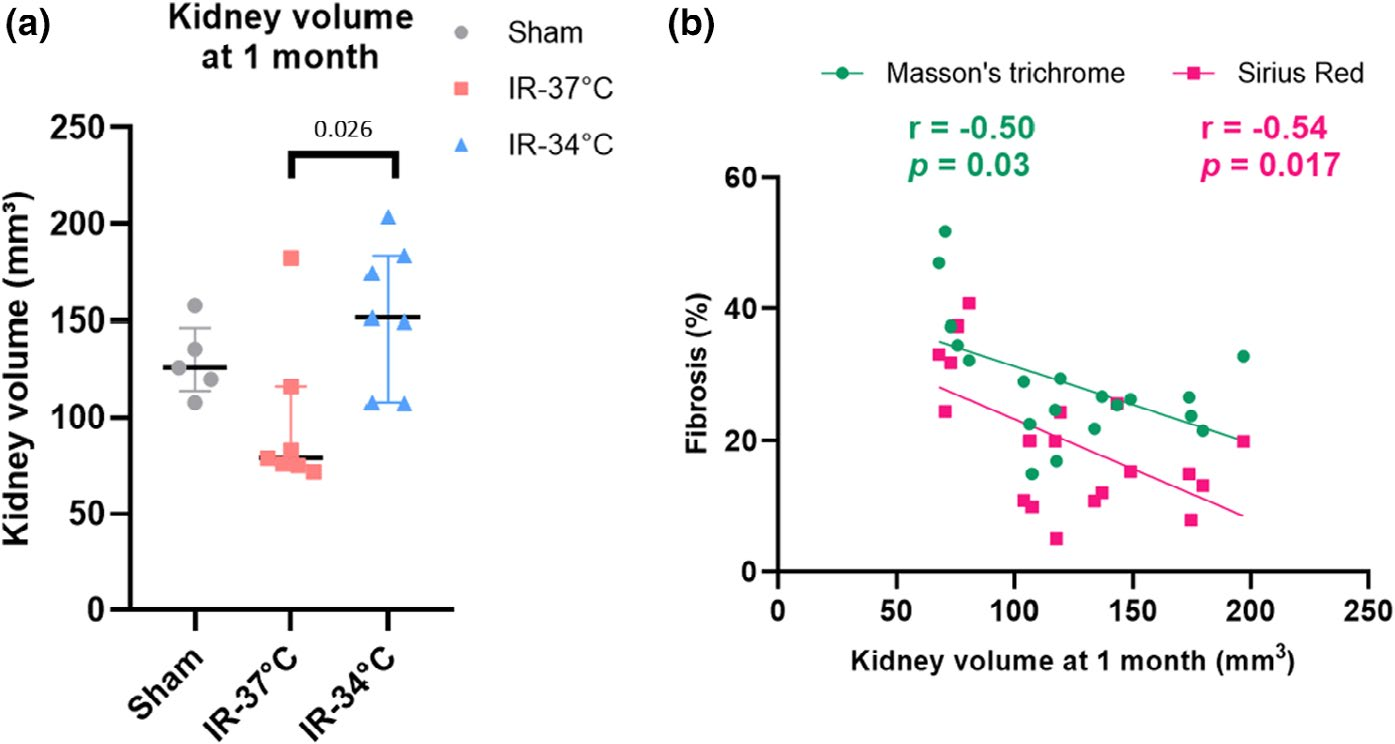
Comparison of absolute ischemic kidney volumes and their correlation with percentage of fibrosis at 1 month. Comparison of absolute (a) ischemic kidney volumes 1 month after reperfusion and correlation (b) between ischemic kidney volume measured at 1 month with 3D reconstruction echography and the percentage of fibrosis on the left kidney quantified on Sirius red (pink) or Masson's trichrome staining (green). Data are presented as median with interquartile ranges and compared with Mann–Whitney test. Correlation is tested by Spearman test.

At 1 month, ischemic kidneys in IR‐37°C were significantly atrophied after normothermic renal ischemia compared to those protected by mTH in IR‐34°C (kidney volume on multiplane 3D reconstruction: 79.03 mm^3^ IQR [75–116] mm^3^ vs. 151.6 mm^3^ IQR [108–184] mm^3^; *p* = 0.026; Figure 4a).

As expected, fibrosis extent and kidney volume measured on 3D reconstruction at 1 month were inversely correlated whatever the method of quantification of fibrosis used: the larger the fibrosis, the smaller the kidney volume (Spearman *R* = –0.54, *p* = 0.017 for Sirius red; *R* = –0.50, *p* = 0.03 for Masson's trichrome; Figure 4b).

#### 3.2.2 Renal PA imaging: Quantification of oxygen content

**FIGURE 5:**
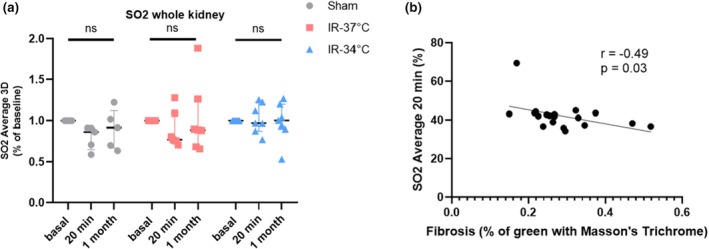
Renal oxygen content in ischemic kidneys during follow up and correlation with fibrosis. Comparisons of renal oxygen content (SO_2_ Average) using PA imaging during follow up in whole kidney (a). Correlation between SO_2_ Average 3D values of the whole kidney 20 min after reperfusion and the percentage of fibrosis evaluated with Masson's trichrome staining 1 month after reperfusion (b). Data are presented as median with interquartile ranges and compared with Wilcoxon test. Correlations are tested by Spearman test.

We showed no significant change in total renal oxygen content on the whole 3D reconstruction of the kidney (SO_2_ Average 3D) after 20 min and 1 month of reperfusion in any group compared to baseline levels (Figure 5a). Cortical, inner medulla, and outer medulla SO2 Average levels were not modified in all groups during follow‐up. Nevertheless, and interestingly, the relative oxygen content measured by PA imaging of the ischemic kidney 20 min after reperfusion correlated significantly with the extent of late fibrosis quantified by Masson trichrome staining at 1 month (R = –0.49; *p* = 0.03; Figure 5b). Similarly, the correlation between Sirius red staining and oxygen content at 20 min tended to be significant (*R* = −0.39; *p* = 0.09). No significant association was observed between glomerular size at 1 month and oxygen content measured after 20 min and 1 month of reperfusion.

#### 3.2.3 Renal CEUS: Quantification of perfusion

**FIGURE 6:**
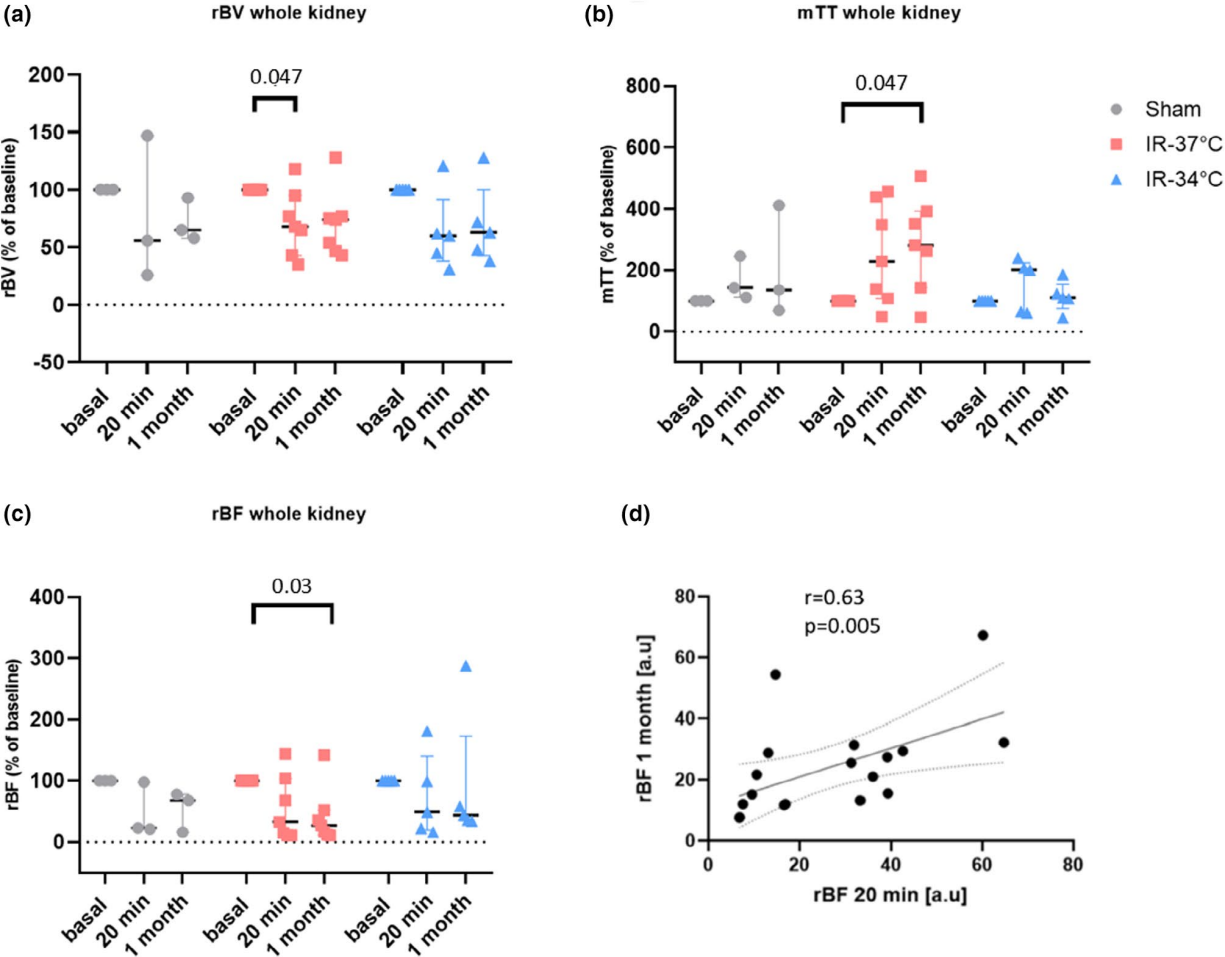
Evolution of CEUS parameters throughout the whole kidney analysis, measured on a 2D transversal section using the Destruction‐Replenishment Perfusion Model (a–c), and correlation between early and late perfusion (d). (a) Relative Blood Volume (rBV), corresponding to the plateau value. Renal rBV decreased in IR 37°C group at 20 min. No significant modification was observed in IR 34°C group during follow up. (b) Renal mean transit time (mTT), that is, the average time that blood takes to transit through the tissue. We observed an increase in mTT at 1 month in IR 37°C group. mTT remained stable in IR 34°C group. (c) Relative Blood Flow (rBV/mTT). Renal rBF was altered at 1 month compared to baseline in IR 37°C group. rBF was not altered during follow up in IR 34°C group. Values are expressed as percentage of baseline. Data are presented as median with interquartile range and compared with Wilcoxon test. (d) Renal rBF measured 20 min after reperfusion showed a correlation with renal rBF values measured at 1 month. Correlation tested by Spearman test. CEUS, contrast‐enhanced ultrasound; rBF, relative blood flow; rBV, relative blood volume; mTT, mean transit time.

Regarding perfusion parameters, our results on whole section of the kidney showed an early deterioration in rBV at 20 min in the IR‐37°C group (68% IQR [43–95]% of baseline; *p* = 0.047) (Figure 6a). In the long term, changes were also observed: 1 month after reperfusion there was a significant decrease in rBF (27% IQR [12–52]% compared to the baseline; *p* = 0.03), linked to a lengthening of mTT (281% IQR [143–392]% of baseline; *p* = 0.047) (Figure 6b,c). Interestingly, the early changes in rBF measured 20 min after reperfusion were significantly correlated with chronic changes of rBF measured 1 month after reperfusion (*R* = 0.63; *p* = 0.0048; Figure 6d), however, no significant association was observed between perfusion parameters and fibrosis.

**FIGURE 7:**
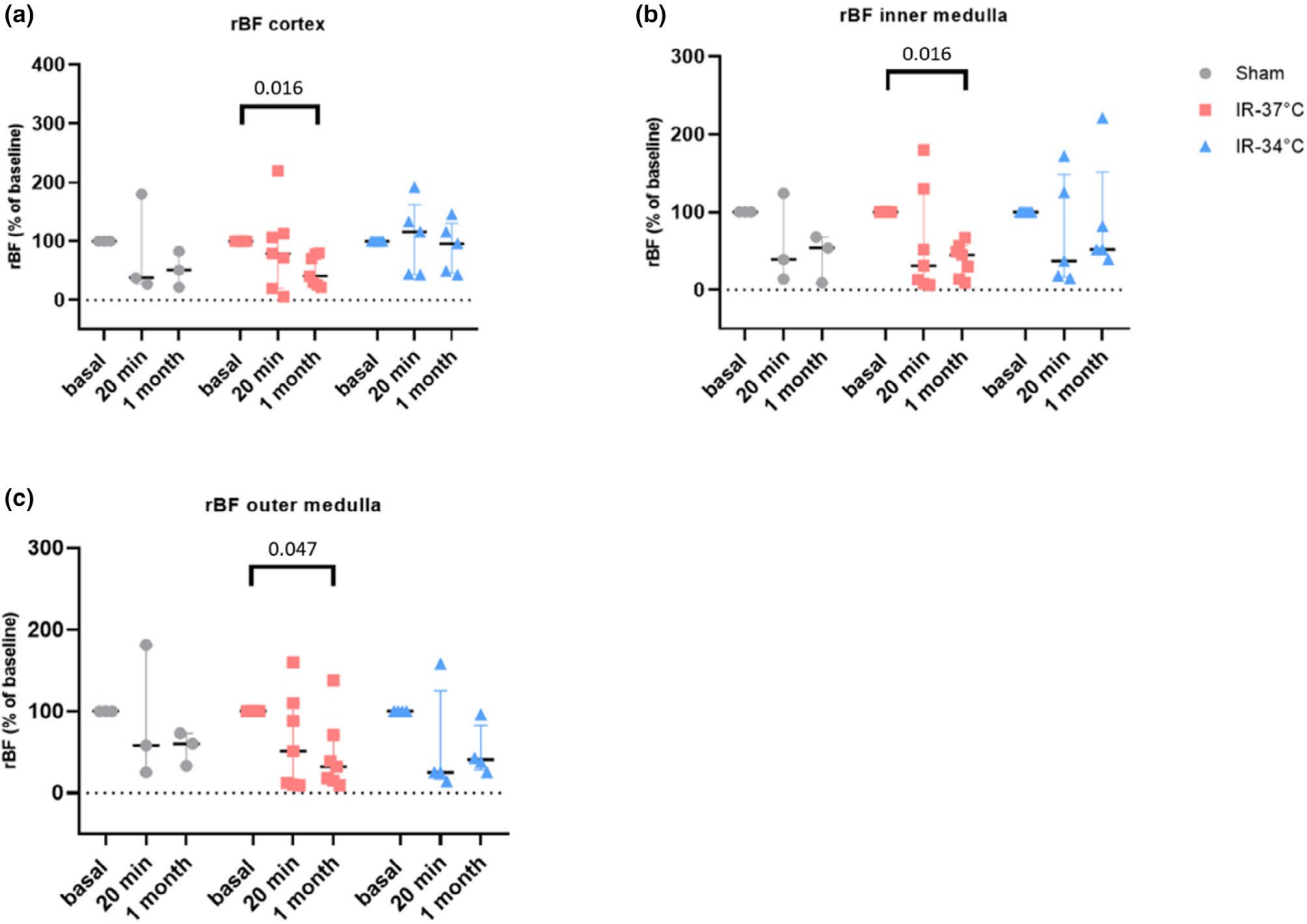
Evolution of renal perfusion (rBF) measured in cortex (a), inner medulla (b), outer medulla (c). rBF, relative blood flow. Values are expressed as percentage of baseline. Data are presented as median with interquartile range and compared with Wilcoxon test.

On regional analysis, that is, with ROI positioned in the cortex (Figure 7a), outer medulla (Figure 7b), and inner medulla (Figure 7c), rBF, rBV, and mTT were altered by IRI in IR‐37°C in each layer of the kidney at 1 month compared to baseline. However, when mTH was applied, we did not observe any significative perfusion alterations in IR‐34°C, neither on whole kidney analysis, nor on regional analysis.

## 4 DISCUSSION

In the present study, we observed that 15 min of unilateral renal ischemia via selective clamping of the left vascular pedicle under normothermic (37°C) conditions in mice resulted in a consistent decline in renal perfusion beginning 20 min after IRI which persisted for up to 1 month after reperfusion. No change in renal oxygen content was detected at these timepoints. This assessment was made possible using two novel imaging tools: renal CEUS and PA imaging. IRI led to histological lesions, such as kidney atrophy, decreased glomerular size, and fibrosis in the downstream kidney. Surprisingly and interestingly, fibrosis was also present in the contralateral untouched kidney. Furthermore, we showed that early changes in renal perfusion as measured by CEUS after renal IRI were correlated with subsequent late impairments after 1 month. Eventually, we observed that mTH can positively impact renal perfusion at both early and late stages and on the development of chronic renal fibrosis. These protective effects of mTH are consistent with previous findings from our group and with clinical studies (Niemann et al., 2015; Schleef et al., 2022).

Renal fibrosis is a critical factor in CKD onset and progression. The traditional kidney biopsy method is an invasive procedure with risks, while it may not accurately reflect the total fibrotic burden due to sampling limitations. It is widely recognized that glomerular filtration rate changes are not accurate enough to detect early damages and early fibrosis after a renal injury. Recent studies have demonstrated that PA imaging can noninvasively quantify kidney fibrosis and assess pretransplant organ quality with high accuracy in settings that mimic human kidney transplantation. This highlights the feasibility of using PA imaging in real‐life applications (Hysi et al., 2020). As this technology continues to evolve, PA imaging could become an integral part of nephrology practice by offering an accurate noninvasive and efficient method for evaluating renal health and guiding therapeutic decisions (Jiang et al., 2023).

It is generally accepted that long duration of ischemia (>25 min) causes severe tubular damage, cell apoptosis, and inflammatory infiltration in the early stage of the disease, leading to chronic kidney fibrosis in the late stage. In contrast, a short duration of ischemia (<20–25 min) induces minimal renal tubular‐interstitial injury that can be fully reversed after the acute phase of kidney injury (Becker et al., 2009; Dong et al., 2019; Thompson et al., 2012). In the present study, ischemia was short and unilateral; however, perfusion remained impaired up to 1 month in three layers of the kidney (cortex, outer medulla, and inner medulla). Additionally, we showed that unilateral IRI surprisingly resulted in histological lesions and increased fibrosis in the contralateral untouched kidney. Following a reduction in renal mass, the remaining kidney is known to undergo adaptive compensatory hypertrophy of both tubules and glomeruli and compensatory glomerular hyperfiltration that may lead to albuminuria and CKD (Helal et al., 2012). However, the underlying pathways mediating fibrosis in this hyperfiltration setting remain unclear but could be due to biomechanical shear stress and podocyte damage (Chagnac et al., 2019; Srivastava et al., 2018; Wagner et al., 2008). These alterations in the remaining kidney can in any case worsen and contribute to CKD development after nephron loss. We also demonstrated that a brief ischemic episode was followed by an early decrease in renal perfusion measured by CEUS, which persisted for 1 month. These results are consistent with those obtained in more severe models of renal ischemia‐reperfusion injury in mice, but still original as no study has yet investigated both early and late timings of CEUS in the same set of experiments (Becker et al., 2009; Cao et al., 2017). All of our observations regarding histological lesions and perfusion alterations following a 15‐min unilateral renal ischemia support the hypothesis, already evoked in some studies, that every minute counts when the renal hilum is clamped (Chen et al., 2022).

We confirmed a significant correlation between the importance of renal hypotrophy and the extent of fibrosis. Therefore, measurement of renal volume in mice using 3D‐reconstruction of renal ultrasound appears to be an interesting proxy tool for indirectly and noninvasively assessing the development of renal fibrosis without the need to sacrifice the animal.

Interestingly, the oxygen content assessed by PA imaging did not differ between groups 20 min or 1 month after reperfusion. Evan et al. suggested that renal cortical and medullary tissue oxygen content can remain stable despite changes in renal blood flow (Evans et al., 2007). An increase in total renal blood flow leads to a proportional increase in glomerular filtration rate, stimulating tubular sodium reabsorption and thus increasing O_2_ consumption. Conversely, lower renal blood flow and glomerular filtration rate would lead to decreased active tubular reabsorption (O'Connor et al., 2006; Rognant et al., 2011). Rognant et al. previously reported that after 4 weeks, no renal hypoxia was detected in the kidney downstream of renal artery stenosis, although this was assessed by BOLD MRI in rats (Rognant et al., 2011). The only study evaluating PA imaging in the setting of IRI‐induced AKI compared a 35‐min clamping of the left renal pedicle (mild renal injury) with a 50‐min clamping (severe renal injury) in mice (Okumura et al., 2018). The authors observed an increase in oxygen content, which peaked at 8 h, and gradually returned to baseline levels thereafter. These results, contradictory to what is reported herein, may be partly explained by different PA imaging timings (ranging from 4 h to 48 h after reperfusion) and a more severe ischemia model than ours. In addition, mouse temperature was not mentioned in the study whereas in our work it was closely monitored and maintained. Nevertheless, we observed a significant negative correlation between renal oxygen content 20 min after reperfusion, and the extent of fibrosis on renal histology at 1 month. This suggests that early measurements of renal oxygen content after IRI could help predict CKD development. This finding is consistent with a previous study that found a correlation between oxygen saturation at 24 h and renal function as assessed by creatinine clearance rate and serum blood urea nitrogen on day 28 (Okumura et al., 2018). Nonetheless, these biomarkers have their limitations and are imperfect to assess precisely renal function, whereas we directly and precisely evaluated renal fibrosis on sections of the whole kidney.

Our study has some limitations. First, the number of animals in each group was small, and we cannot exclude a lack of statistical power to detect small changes in the measured parameter, such as oxygen content. Nevertheless, post hoc calculations showed that the study was powered to detect a reduction of SO_2_ greater than 25%–30% (expected to have physiological consequences) during follow‐up. Second, ROI in the inner and outer medulla were selected at the operator's discretion, although operators were two nephrologists with knowledge of renal anatomy and physiology, and this was aided by histological analysis. Third, the timings of the evaluations at 20 min and 1 month were based on previous work by our group (Schleef et al., 2022). These time points were chosen to describe the very early impact of IRI, when nephroprotective strategies might already have an impact (Lemoine et al., 2015), as well as well‐established late chronic lesions, which can also be improved by nephroprotection (Schleef et al., 2022). Nevertheless, we cannot rule out the possibility that the lack of significance of the changes in renal oxygen content is also partly explained by timing of these measurements. The only PA imaging study on a similar model of renal IRI in mice observed differences in oxygen content between 4 h (their earliest timepoint) and 24 h of reperfusion, but not at 48 h (their latest timepoint). Fourth, a lack of sensitivity in our PA measurements cannot be excluded, and perhaps our experimental conditions did not sufficiently modify oxygen content measurements to reveal differences between our groups, although it was enough to induce chronic fibrosis. Furthermore, the use of inhaled anesthesia could have influenced the results due to its effect on renal vasculature (Franzén et al., 2022) In addition, we used C57BL6 mice whose black fur can generate significant imaging artifacts on PA imaging that might reduce sensitivity, although we carefully removed all hair around the imaging site and adjusted imaging parameters as advised to ensure reliable measurements (Dai et al., 2024). Fifth, we used only male mice to ensure better comparability of the animals, but this can limit the generalizability of these results. Finally, we only showed statistical correlation between the parameters, but we did not demonstrate causality, and further studies are required to confirm these results in other models and settings.

## 5 CONCLUSION

Renal CEUS and PA imaging of the kidneys are two promising tools for the early assessment of the impact of renal IRI in mouse models. In mice, a brief period of normothermic unilateral renal ischemia injury resulted in an early decline in perfusion monitored at 20 min, which persisted for up to 1 month after reperfusion. This was accompanied by increased fibrosis and atrophy in the downstream kidney. PA imaging early measurements at 20 min also correlated with subsequent fibrosis at 1 month. The effects of a nephroprotective intervention such as mTH could also be detected. Emerging ultrasound techniques, such as CEUS and PA imaging, expand the capabilities to obtain renal anatomical and functional information noninvasively.

## AUTHOR CONTRIBUTIONS

**Maxime Schleef:** Conceptualization; formal analysis; investigation; methodology. **Corentin Tournebize:** Data curation; formal analysis; investigation. **Christelle Leon:** Investigation. **Bruno Pillot:** Investigation. **Stéphanie Chanon:** Investigation. **Gabriel Bidaux:** Project administration; resources. **Laurent Juillard:** Funding acquisition. **Fitsum Guebre‐Egziabher:** Funding acquisition. **Delphine Baetz:** Conceptualization; methodology; supervision; validation. **Sandrine Lemoine:** Conceptualization; methodology; supervision; validation.

## FUNDING INFORMATION

This work was supported by Leducq Transatlantic Network of Excellence “Targeting Mitochondria to Treat Heart Disease ‘MitoCardia” grant 16 CVD 04 and ANR (Agence Nationale pour la Recherche) ANR‐23‐CE18‐0006‐02. The funding sources had no involvement in study design; in the collection, analysis, and interpretation of data; in the writing of the report; and in the decision to submit the article for publication.

## CONFLICT OF INTEREST STATEMENT

The authors have no conflict of interest to declare.

## ETHICS STATEMENT

All procedures conducted in this study were approved by the Ethics Committee (Claude Bernard Lyon 1 University, CEEA‐55, no DR2019‐09) and conducted in accordance with French and European Law.

## Supporting information


Figures S1–S3.

## Data Availability

The data that support the findings of this study are available from the corresponding author, upon reasonable request.
