# Supplementary material for: Contrast‐enhanced ultrasound and photoacoustic imaging to assess early and late renal ischemia‐reperfusion injuries in male mice
Source: Physiol Rep. 2026 Jun 18;14(12):e70973. doi: 10.14814/phy2.70973 (PMC13276675; doi:10.14814/phy2.70973)
Supplement: Supplementary file 1 — Figures S1–S3. [file PHY2-14-e70973-s001.docx]

**SUPPLEMENTAL**

**Figure Legends:**

**Figure S1. Example of fibrosis automatic quantification using Fidji software on a Sirius red coloration.**

(1) Original 4-μm kidney section stained with Sirius Red, followed by (2) color deconvolution (left panel, pixels containing a red component; right panel, any colored pixel), and (3) quantification of the pixels reaching a threshold for the deconvoluted color (colored in black by the software), i.e. deconvoluted red pixels (left panel) and any color pixels (right panel) representing the whole kidney biopsy. The ratio of red pixels to whole kidney represents fibrosis, expressed as a percentage.

**Figure S2. Example of CEUS imaging and mathematical models underlying the "destruction-replenishment" mode.**

(A) Above, example of CEUS imaging with ROI selection. Purple = inner medulla; blue = outer medulla; yellow = cortex; green = whole kidney. The selected regions were maintained (placed manually) on consecutive images (basal, 20min and 1 month after 15 min ischemia-reperfusion). Bottom, A brief emission of high-intensity ultrasound destroys microbubbles.

**Figure S3. Example of PA imaging at 20min with regions of interest selection on parametric maps and on B-mode images.** Sky blue = inner medulla, SO2 average 35.11%; green = outer medulla, SO2 average 41.47%; navy blue = cortex, SO2 average 44.88%; orange = whole kidney, SO2 average 41.07%. The selected regions were maintained (placed manually) on consecutive images (basal, 20min and 1 month after 15 min ischemia-reperfusion).

**
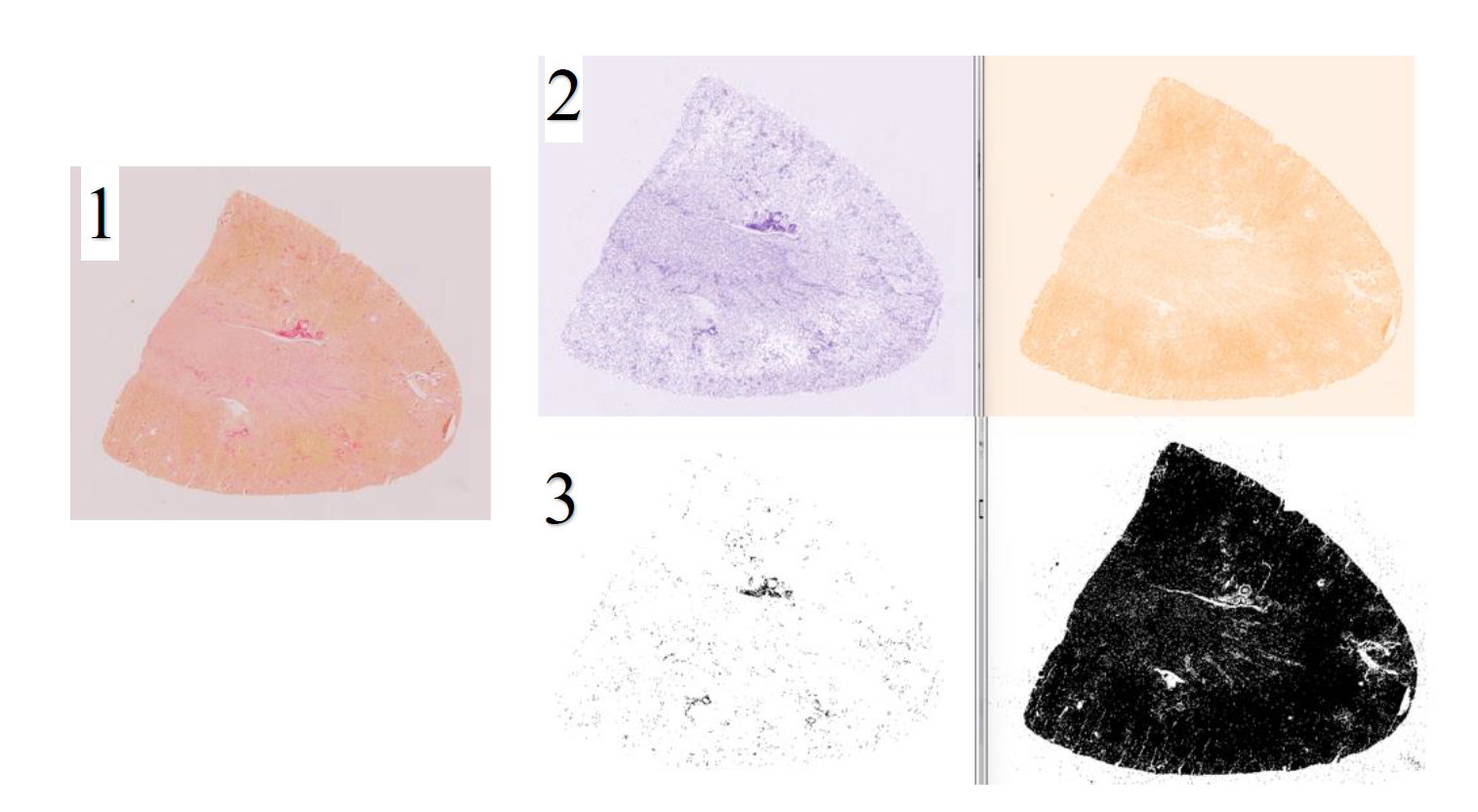
**

**Figure S1.**


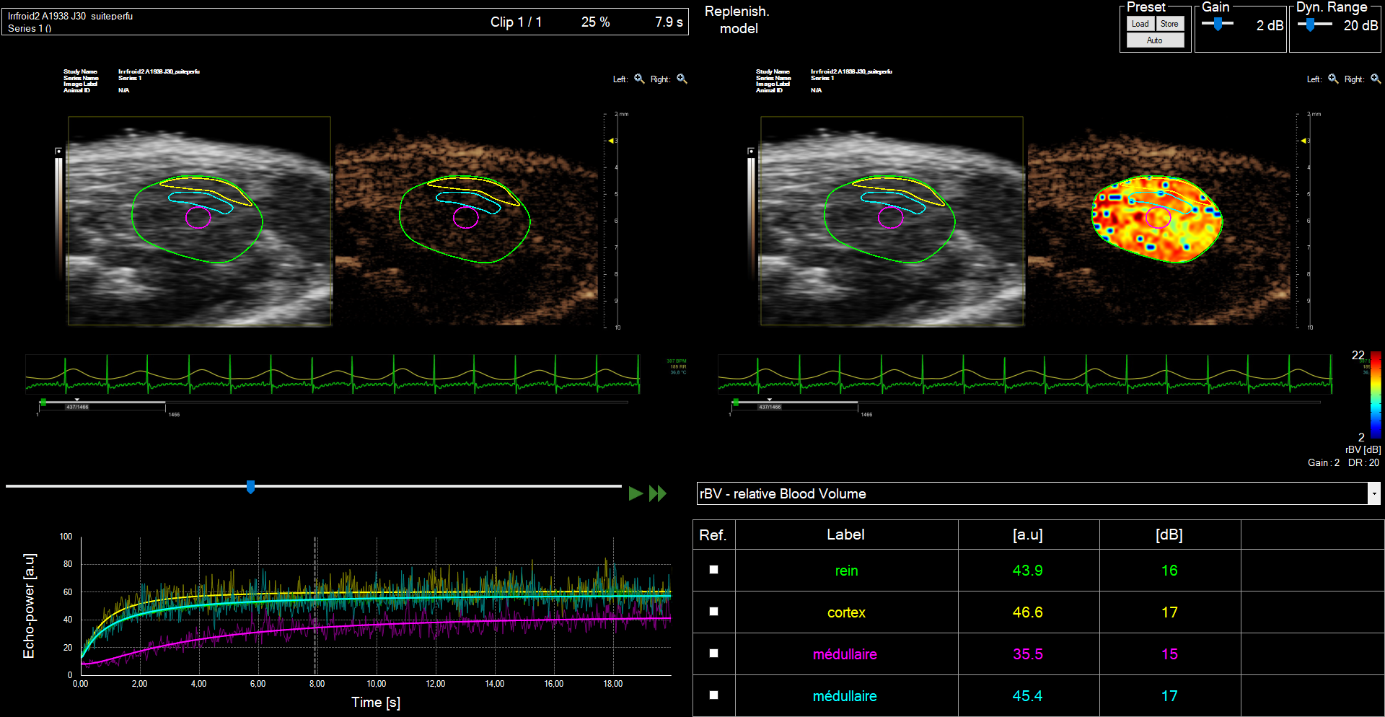


**B**

**A**


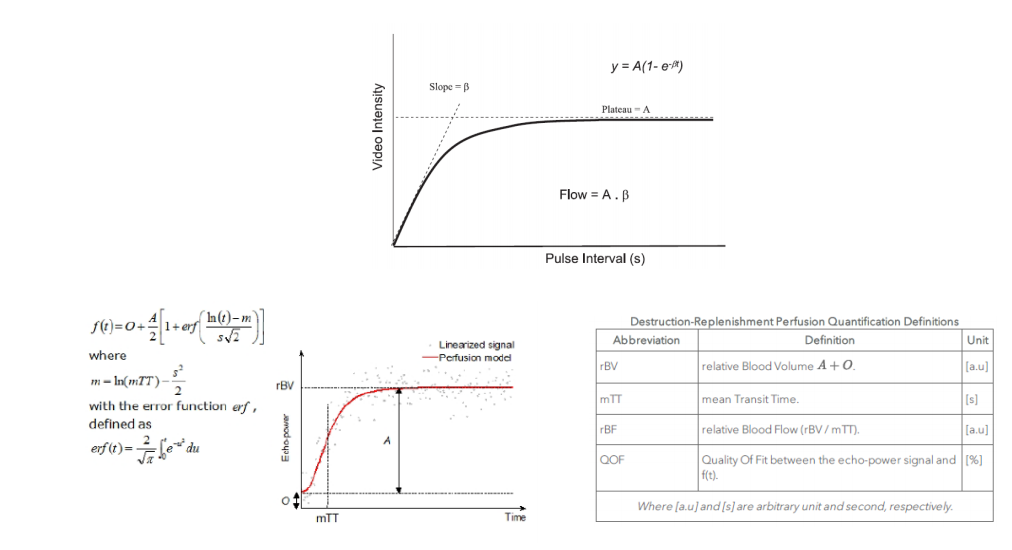


**Figure S2.**

**
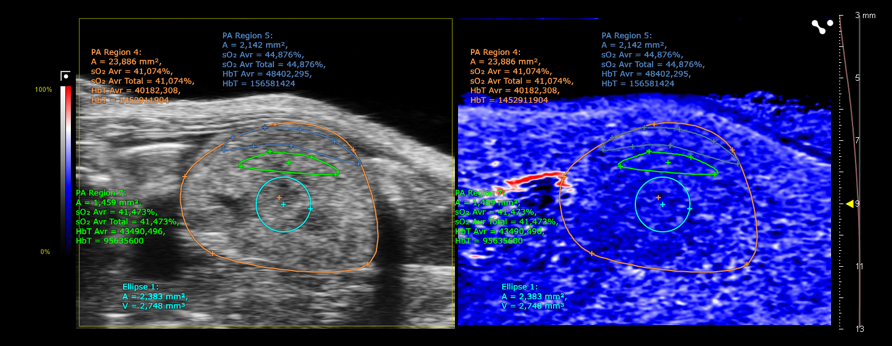
**

**Figure S3.**
